# Right Ventricular Myocardial Infarction Complicated by Cardiac Arrest: Utilization of Extracorporeal Membrane Oxygenation

**DOI:** 10.1155/2022/2462781

**Published:** 2022-02-17

**Authors:** Alisha Alabre-Bonsu, Saurav Uppal, Ernest L. Mazzaferri, Konstantinos Dean Boudoulas

**Affiliations:** Division of Cardiovascular Medicine, The Ohio State University, Columbus, Ohio, USA

## Abstract

A 44-year-old male with an out-of-hospital cardiac arrest due to an acute left ventricular (LV) inferoposterior wall myocardial infarction (MI) involving the right ventricle (RV) is presented. This case highlights the challenges in the management of patients with cardiac arrest, indications for use of ventricular assist devices, potential effects of LV assist devices on the RV in the setting of RV MI, and culprit versus complete coronary artery revascularization in these patients.

## 1. Introduction

Combined left ventricular (LV) and right ventricular (RV) myocardial infarction (MI) is associated with greater morbidity and mortality as compared to an isolated LV MI. RV dilatation and marked decrease in RV stroke volume may occur in RV MI leading to systemic hypotension, tissue hypoperfusion, and cardiogenic shock. Further, cardiac arrest is not uncommon during the acute phase of MI leading to a poor prognosis [1]. While LV assist devices have been shown to be effective in the management of LV failure due to an acute MI, in cases of combined RV and LV MI, these devices may increase RV work due to increased volume return to the RV resulting in deterioration of its function [2]. A 44-year-old male with an out-of-hospital cardiac arrest due to an acute LV inferoposterior wall MI associated with a RV MI is presented, and current management of cardiac arrest in these patients is briefly discussed.

## 2. Case Presentation

A 44-year-old man without a significant past medical history developed chest pain for approximately one hour while at his office prior to being found unconscious by one of his colleagues. Bystander cardiopulmonary resuscitation (CPR) was not performed at that time. Emergency medical services (EMS) arrived within 5 minutes and initiated advanced cardiovascular life support (ACLS). The patient was intubated and underwent multiple unsuccessful defibrillation attempts for ventricular fibrillation. Since return of spontaneous circulation (ROSC) could not be obtained, an automated CPR device (LUCAS chest compression system, Stryker Medical, Portage, Michigan) was placed and initiated at 100 beats per minute. The patient was emergently transported directly to the cardiac catheterization laboratory (CCL) at The Ohio State University as part of an extracorporeal cardiopulmonary resuscitation (ECPR) program that utilizes venoarterial extracorporeal membrane oxygenation (ECMO) as an adjunct to CPR for out-of-hospital cardiac arrest due to refractory ventricular tachycardia/fibrillation despite three defibrillation attempts [3]. Upon arrival to CCL, the patient was found to be in persistent ventricular fibrillation; thus, he was defibrillated once again; this time, ROSC was achieved.

**Figure 1:**
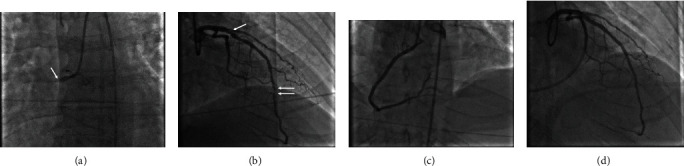
(a) Diagnostic arteriogram demonstrating acute complete thrombotic occlusion in the proximal right coronary artery (arrow) representing the culprit lesion and (b) 90% stenosis in the proximal left anterior descending (LAD) coronary artery (arrow) and 95% stenosis in the mid LAD (double arrow). (c) Coronary arteriogram post-percutaneous coronary intervention (PCI) of the RCA with placement of drug eluting stents with restoration of coronary blood flow. (d) Coronary arteriogram post-PCI of the proximal and mid LAD with placement of drug eluting stents.

**Figure 2:**
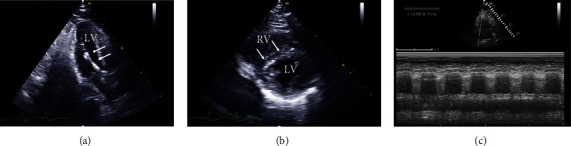
(a) Transthoracic echocardiogram (TTE) 3-chamber showing the Impella CP percutaneous left ventricular assist device (arrows) within the left ventricle (LV). (b) TTE short axis view demonstrating septal flattening (arrows) indicating right ventricular (RV) pressure/volume overload; the RV is dilated as well. (c) TTE M-mode of the tricuspid annulus showing decreased (dashed line) tricuspid annular plane systolic excursion (TAPSE) of 0.73 cm suggesting RV dysfunction.

Coronary arteriography demonstrated complete thrombotic occlusion in the proximal right coronary artery (RCA; Figure 1(a)) and 90% stenosis in the proximal and 95% stenosis in the mid left anterior descending (LAD) coronary artery (Figure 1(b)). Percutaneous coronary intervention (PCI) with the placement of drug eluting stents in the RCA (culprit lesion) was performed restoring coronary blood flow (Figure 1(c)). PCI was not performed in the LAD stenoses. After PCI and despite an epinephrine infusion, the patient remained in cardiogenic shock with a systemic arterial pressure of 75/47 mmHg. At that time, a left and right heart catheterization demonstrated LV end diastolic pressure (LVEDP) 18 mmHg, right atrial pressure (RAP) 14 mmHg (mean), pulmonary artery pressure 28/16 mmHg, pulmonary capillary wedge pressure (PCWP) 16 mmHg (mean), and cardiac index (CI) 1.1 L/min/m^2^. The pulmonary artery pulsatility index (PAPi) (pulmonary artery pulse pressure over RAP) when <1.0 is considered an index of RV failure in acute MI [2]. A ratio of RAP to PCWP >0.86 is also considered an index of RV failure in acute MI [2]. The PAPi in this case was 0.9, and RAP/PCWP was 0.87; both of these indices were borderline for RV failure. Since the patient was in cardiogenic shock with an elevated LVEDP and low CI with borderline values for RV failure, the decision at that time was to support the LV with an Impella CP percutaneous LV assist device (Abiomed, Inc., Danvers, Massachusetts) that was placed via the femoral artery (Figure 2(a)). Impella was chosen due to its ease and quickness of implantation compared to ECMO, which can be more cumbersome to set-up, implant, and initiate. The patient was then admitted to the cardiovascular intensive care unit (CVICU) for further management.

**Figure 3:**
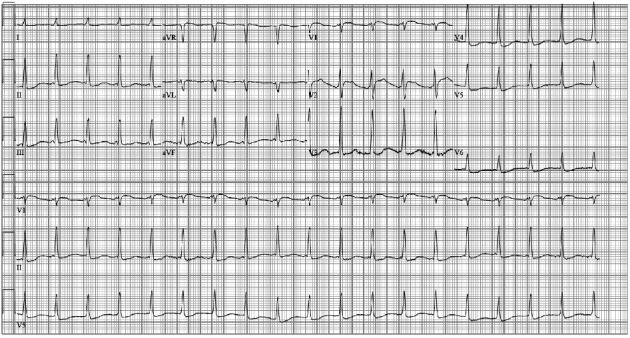
Electrocardiogram obtained upon arrival to the cardiovascular intensive care unit demonstrating supraventricular tachycardia (ectopic atrial versus junctional tachycardia), early R-wave transition in the precordial leads, diffuse ST and T wave changes, and QT prolongation most likely secondary to myocardial injury/ischemia and metabolic abnormalities due to cardiac arrest.

Upon arrival to the CVICU, an electrocardiogram (ECG) was obtained that demonstrated supraventricular tachycardia (ectopic atrial versus junctional tachycardia), early R-wave transition in the precordial leads, diffuse ST and T wave changes, and QT prolongation most likely secondary to myocardial injury/ischemia and metabolic abnormalities due to cardiac arrest (Figure 3). Shortly after his arrival to the CVICU, the patient had a recurrent ventricular fibrillation in which he underwent ACLS and one defibrillation achieving ROSC. At that time, the lactate (14 mmol/L), serum creatinine (2.1 mg/dL), alanine aminotransferase (183 U/L), and aspartate aminotransferase (348 U/L) were elevated. A transthoracic echocardiogram (TTE) demonstrated severe LV systolic dysfunction (ejection fraction approximately 20%), RV enlargement with moderately severe RV systolic dysfunction, and septal flattening suggestive of RV volume/pressure overload (Figures 2(b) and 2(c)). Due to recurrent ventricular fibrillation and persistent cardiogenic shock despite intravenous pressor and Impella support, the decision was made to take the patient back to the CCL for placement of ECMO for escalated hemodynamic support and PCI of the LAD. Successful PCI of the proximal and mid LAD stenoses with placement of drug eluting stents was performed (Figure 1(d)). In addition, the Impella was removed, and the patient was placed on venoarterial ECMO through cannulas placed in the femoral artery and vein.

The patient had a prolonged hospital stay that was further complicated by persistent acute kidney injury requiring hemodialysis. Support with dobutamine infusion was required. Eventually, ECMO support was slowly weaned over several days, and on hospital day 5, the ECMO cannulas were successfully removed in the operative room. Following removal of ECMO, the patient did require additional inotropic support; thus, a milrinone infusion was initiated along with the preexisting dobutamine infusion. Milrinone was able to be weaned off after 48 hours. The dobutamine infusion was slowly weaned off after a total therapy of 13 days. A TTE was repeated 2 weeks after his initial presentation that demonstrated almost full recovery of LV (ejection fraction approximately 55%) and RV function. Physical therapy was performed initially at the bedside on day 10 of hospitalization, and by day 16, the patient was ambulating the hospital hallway without difficulty. The patient was evaluated by the electrophysiologist for possible placement of an implantable cardioverter defibrillator (ICD) given presentation of ventricular fibrillation cardiac arrest; however, this was deferred due to successful coronary artery revascularization and recovery of LV systolic function. Optimal guideline directed medical therapy for heart failure was initiated and slowly titrated over the course of patient's hospitalization including carvedilol 25 mg twice daily, hydralazine 50 mg every 8 hours, and isosorbide mononitrate 60 mg daily. Renin angiotensin aldosterone system inhibitor was not initiated due to acute renal failure. In addition, the patient was taking aspirin 81 mg daily and clopidogrel 75 mg daily due to coronary artery stent placement. The patient was discharged to home on day 20 of hospitalization with full neurologic capacity and with outpatient hemodialysis and cardiology follow-up.

The patient was evaluated in cardiology clinic 2 weeks after hospital discharge. He was asymptomatic and was attending cardiac rehabilitation. His acute renal failure had improved (serum creatinine 1.8 mg/dL) and he no longer required hemodialysis. Eighteen months after hospital discharge, the patient remained asymptomatic and was training for a 5-kilometer race.

## 3. Discussion

Despite recent medical progress, the prognosis in cardiac arrest due to MI remains poor and the management is quite challenging. This case highlights the importance of aggressive and timely therapy in this group of patients [1]. Emergent coronary artery revascularization of the culprit lesion is the mainstay of therapy, and fluid resuscitation may be required in cases of RV MI. The high RV preload, however, needed to maintain a cardiac output may result in an increase in RV work in the setting of an ischemic myocardium [1, 2]. In addition, RV volume overload can lead to leftward shifting of the LV septum resulting in a further decrease in LV stroke volume and cardiac output [4].

The diagnosis and the severity of RV dysfunction that leads to failure can be established by echocardiography and hemodynamic assessment (Figures 2(b) and 2(c)). Echocardiogram will demonstrate a dilated and hypokinetic RV with diminished ejection fraction. Right heart catheterization can be utilized to determine various hemodynamic parameters including PAPi (<1.0) and ratio of RAP to PCWP (>0.86) that suggest RV failure in the setting of an acute MI [2]. Limitations including accuracy of these pressure-related indices, mostly based on recordings using fluid-filled catheters, should be acknowledged [1]. Since the patient was in cardiogenic shock with an elevated LV end diastolic pressure and low CI, and borderline values for RV failure, the decision made at that time was to support the LV with placement of an Impella percutaneous LV assist device. Shortly after his arrival in the CVICU, the patient developed another episode of ventricular fibrillation. If this was partially related to an increase in cardiac output after the placement of the LV assist device that resulted in an increase in RV work due to an increase volume return to the RV cannot be proven or disproven. Likewise, if the last episode of ventricular fibrillation could have been avoided if ECMO was initially placed to provide full cardiac support not only to the LV but also to the RV, cannot be proven after the fact; however, such an approach in patients with LV and RV failure should be considered.

ECMO is effective in providing hemodynamic support for individuals with refractory cardiogenic shock due to LV and/or RV failure [5, 6]. Pulmonary vasodilators may have some utility in supporting acute RV failure [7]. In addition, RV assist devices can be utilized for RV support [2]. However, ECMO was eventually chosen in this particular case due to the need to support both ventricles. While ECMO can provide hemodynamic support in LV failure, it is important to be aware of the LVEDP, as an increase in LVEDP may result in an increase in pulmonary edema, increase in myocardial oxygen demand, and vulnerability to ischemia-mediated necrosis [5, 8]. Interventions that may assist in decreasing an elevated LVEDP while on EMCO include use of inotropes, vasodilators, intra-aortic balloon pump, and percutaneous left ventricular assist devices [6, 9]. Since ECMO is often used as a bridge to recovery in patients with reversible cardiac injury, it is important when initiating ECMO to assess which patients may derive the most benefit and likelihood of recovery; various risk scores are available to assist with this; however, nothing replaces sound clinical judgement and experience [5].

It is important to be aware that after placement of a LV assist device, RV failure has been reported in approximately 13-44% of patients [10]. Reassessing hemodynamic parameters with a right heart catheterization, as described previously, after placement of a LV assist device should be performed when there is concern for RV failure. In addition, several echocardiographic variables such as RV fractional area change, RV/LV diameter ratio, RV free wall longitudinal strain by velocity vector imaging, and tricuspid annular plane systolic excursion (TAPSE; Figure 2(c)) have been found to be useful in predicting RV failure after placement of a LV assist device [10].

Another dilemma in this case was the decision to either only perform PCI of the culprit lesion or pursue complete coronary artery revascularization during the index procedure. There are several studies including the PRAMI, COMPLETE, and DANAMI-3 PRIMULTI trials that demonstrated improved cardiovascular outcomes with complete coronary artery revascularization immediately or staged within the index hospitalization for patients presenting with ST-elevation myocardial infarction (STEMI) without cardiogenic shock [11–13]. Conversely, the CULPRIT SHOCK trial demonstrated that patients with an acute MI and cardiogenic shock had a significantly lower risk of mortality and required less often renal-replacement therapy at 30 days when PCI was only performed on the culprit lesion as compared to immediate multivessel PCI, and there was no significant difference in mortality between the two groups at 1-year follow-up [14]. There is limited data, however, to guide management of patients who present with cardiac arrest and cardiogenic shock who are found to have multivessel coronary artery disease. “Individualization” is essential, as in these cases, one size does not fit all. In our case, the initial revascularization was targeted towards the culprit RCA lesion. It is unclear as to whether or not upfront PCI to the LAD could have prevented further decompensation in this patient. Further, it is unclear if acute renal failure could have been avoided or if the outcome of the patient could have been different if LAD revascularization was deferred for a later time.

## 4. Conclusion

This case highlights the challenges in the management of a patient with cardiac arrest even after achieving ROSC. Understanding the pathophysiology of LV and RV infarction and their interrelationship is critical in order to provide optimal care. It is also important to understand the potential effects on the RV in the setting of RV MI when unloading the LV with a LV assist device and the potential benefits of ECMO. In addition, the risk of complete revascularization due to longer procedural times and increase use of contrast material should always be taken into consideration. It is difficult to establish fixed rules for these critically ill and unstable patients in which individualized therapy based on knowledge, clinical experience, and common sense should be applied.

## Data Availability

No data was used to support this study.
